# Coronary Computed Tomography Angiography Takes the Center Stage and Here is Why

**DOI:** 10.5935/abc.20190003

**Published:** 2019-01

**Authors:** Illan Gottlieb, Marcio Sommer Bittencourt, Carlos Eduardo Rochitte, João L. Cavalcante

**Affiliations:** 1 Casa de Saúde São José - Radiologia, Rio de Janeiro, RJ - Brazil; 2 Universidade de São Paulo - Hospital Universitário de São Paulo, São Paulo, SP - Brazil; 3 Universidade de São Paulo - Faculdade de Medicina Hospital das Clinicas Instituto do Coração, São Paulo, SP - Brazil; 4 Minneapolis Heart Institute at Abbott Northwestern Hospital, Minneapolis, Minnesota - USA

**Keywords:** Coronary Artery Disease/diagnostic imaging, Coronary Artery Disease/prevention and control, Coronary Artery Disease/physiopathology, Computed Tomography Angiography, Tomography, X-Ray Computed

Max Planck once said that “A new scientific truth does not triumph by convincing its opponents and making them see the light, but rather because its opponents eventually die, and a new generation grows up that is familiar with it.” In its beginnings, coronary computed tomography angiography (CCTA) was accused of having too low accuracy for the diagnosis of obstructive coronary artery disease (CAD) to be used in clinical practice. Over the last decade, major technical developments such as larger axial coverage (from 2 cm to 16 cm) and improved temporal resolution, have enabled CCTA to become by far the most accurate non-invasive imaging method for diagnosis of obstructive CAD, with sensitivity and specificity of approximately 95% and 90%, respectively.^1^

Then CCTA was burdened with the accusation of exposing patients to radiation doses so high, that warranted some society guidelines to specifically point this out and limit its use. At that time, CCTA exposed patients to doses ranging from 20 to 25 mSV, while triphasic abdomen CT exposed patients to 30 to 40 mSv and scintigraphic myocardial perfusion studies with Thallium used up to 40 mSv. In 2018, radiation exposure from CCTA dropped to well below 5 mSv (most advanced clinical centers use much less), a fraction of the dose used in myocardial perfusion studies with MIBI tetrophosmin.^2^ Then the cost-effectiveness wave came with societies rightfully demanding proof that CCTA offered more value at an acceptable cost compared to other imaging modalities, and CCTA once again proved to be more cost-effective than other modalities.^3^ Although one hardly finds cost-effectiveness studies comparing nuclear scans with ECG treadmill tests, providing better diagnosis performance is not enough anymore. More recently, this strategy has even been put into challenge in large randomized clinical trials comparing CCTA with the standard of care in the investigation of suspected CAD both in the acute and in the outpatient settings.^4^^-^^9^

But then CCTA adoption had to face another hurdle. People started demanding that CCTA, a diagnostic study, should demonstrate that it would alter clinical outcomes. Let’s stop here for a moment: a diagnostic study makes the diagnosis. It does not provide the cure, but it could lead to changes in therapy which could eventually lead to improved outcomes. As such, although a CCTA study is not therapeutic, it could guide and inform therapeutic decisions. Measuring blood pressure was never proven to alter clinical outcomes, treatment did. The same with cholesterol measurements, ischemia testing and resting ECG recordings. And yet, everybody has always rightly assumed that diagnosis is a fundamental part of sound medical practice and an angular stone of clinical management. Cardiovascular disease, predominantly in the form of atherosclerosis and hypertension, starts as early as 30 or 40 years, silently progressing across the years to finally kill around one-third of the adult population in the developed world. The conventional strategy “to sit and wait” until patients present with symptoms certainly misses the golden period of the early disease, when treatment is much more efficient and less expensive. Early detection and diagnosis of atherosclerosis using CCTA, might lead to significant downstream changes which could consequently improve outcomes.

Despite those initial criticisms and the sceptical view of the use of CCTA in the investigation of suspected CAD, the evidence supporting its clinical use has been steadily increasing over the years. From the initial studies defining the technical feasibility and accuracy of CCTA, followed by the development of techniques aimed at reducing radiation dose and improving imaging quality, CCTA has evolved to be part of the routine armamentarium for the investigation of suspected CAD. More recent evidence has led a wide variety of interpretations, as CCTA lead to an increase in the diagnosis of CAD, accompanied by a 31% reduction in the rate of myocardial infarction, while also being associated with a modest increase in the use of invasive coronary angiography (ICA) and revascularization, according to a recent meta-analysis.^6^ The potential impact of those findings have recently been enhanced by the publication of the 5 years follow up data of the SCOT-HEART trial.^5^

The SCOT-HEART study randomized more than 4,000 individuals with symptoms suggestive of CAD to usual care (UC), which includes the use of stress treadmill testing or nuclear perfusion studies, versus UC combined with CCTA. In their initial report in 2015,^9^ the authors demonstrated that the use of CCTA led to change in the initial clinical diagnosis in more than one in every four patients. It is particularly interesting to note that this was driven by an increase in the prevalence and certainty for the diagnosis of CAD overall, but also by an increase in certainty with a decrease in the prevalence of angina due to CAD. Those changes in diagnosis also led to meaningful changes in the management of this population.

When compared to the UC arm, the addition of CCTA resulted in a changed in the use of additional testing in 15% of the population (vs. 1% in the UC), and the use of medications in 23% (vs. 5% in the UC, p < 0.001) for both. It is particularly important to dissect those changes to appropriately understand the impact of CCTA on the initial management of this population. The additional information provided by the CCTA improved diagnostic certainty both due to the increase and decrease in the likelihood of disease after a positive and negative CCTA result, respectively. Thus, for the downstream use of diagnostic testing in the UC group, upon the 6-week return visit, there were overall 6 additional stress imaging tests, 8 ICAs performed and only one ICA cancelled. On the other hand, in the UC + CCTA group there were 121 stress imaging tests, 29 ICA cancelled, 5 additional stress imaging tests and 94 ICA tests performed. Collectively, this suggests that these differences in downstream additional testing were the result of additional information provided by the CCTA.

A similar pattern of change was also noted on the use of medications in both groups. In the UC there was minimal cancellation of preventive and antianginal medication (0.4% and 0.3% of patients, respectively), but a significant increase in its use (4.1% and 0.5% patients, respectively). On the other hand, a much larger shift in the use of medications was noted in the UC + CCTA arm, in both directions and both for preventive and antianginal medications. Those medications were started in 14.1% and 4.0% individuals, respectively, and stopped in 3.7% and 5.4% individuals, respectively. It is also worth noting that those results might underestimate the true changes in management, as the authors did not capture changes in medication dose/intensity, nor were any documentation of changes in non-pharmacological therapy available. Importantly, the changes in revascularization did not reach statistical significance, though they were numerically more frequent in the UC + coronary CTA arm (11.2 vs. 9.7%, p = 0.06).

It is important to highlight that even this extent of detail in medication change during the course of the SCOT-HEART study still overly simplifies its potential impact in event reduction. The actual change in therapy cannot be fully appreciated simple by counting the number of individuals who underwent changes in prescription without qualitative information on this population. Individuals in whom therapy was reduced were, in general, individuals with no or mild coronary atherosclerosis, whereas individuals in whom therapy was increased were individuals with more extensive and severe CAD. Thus, therapy was targeted and individuals more likely to derive benefit.

Despite those changes in management, the initial publication of SCOT-HEART left some gaps in the understanding of the impact of those findings, as both groups had similar improvement in the angina frequency and stability after 6 weeks, and the changes in hard outcomes did not reach formal statistical significance despite the almost 40% reduction in events noted in the study. Those results were questioned even further as the concurrent U.S. based study PROMISE, published simultaneously, showed no difference in outcomes in individuals with suspected CAD investigated with coronary CTA vs. UC, which in the U.S. was mostly based on imaging stress testing. However, several differences between the two studies justify differences in the findings, from differences in patient population, age, sex, symptoms, as well as pre-test probability of disease. Additionally, differences in medication changes during the follow up were noted. While care after testing was left at the discretion of the attending physician in both trials, SCOT-HEART had a structured protocol to recommend preventive medical therapy to individuals with non-obstructive CAD on the coronary CTA, whereas PROMISE did not make any recommendations.^10^

The trend in outcomes reduction documented in SCOT-HEART was further replicated in a meta-analysis and in a large Danish registry.^5^^,^^11^ In both studies an increase in revascularization was also noted, and the Danish study also demonstrated that a concurrent increase in the use of preventive therapy (aspirin and statin) was noted.

Yet, none of those results led to nearly as much repercussion on the topic as the recent publication of the 5-year follow up of the SCOT-HEART.^12^ In the longer term follow up of the same cohort of patients, several important differences need to be highlighted. First, with the larger number of events, there is a higher precision on the estimates of benefit, and a 40% reduction in the rate of coronary heart disease death or myocardial infarction (p < 0.004) was now documented. A second important finding of the study is the fact that the initial increase in the rate of ICA and revascularizations was no longer seen at 5 years. While the rate of ICA was 23.6% in the UC + coronary CTA arm, it was 24.2% in the UC arm (hazard ratio: 1.00, 95% confidence interval 0.88 - 1.13). This fact occurred as the UC arm had higher rates of ICA and revascularizations after the initial evaluation. Using a landmark analysis with a starting point at 12 months, the UC + CCTA arm had a 30% reduction in the rate of ICA through 5 years and a 40% reduction in late revascularizations when compared to UC.

Another relevant aspect of SCOT-HEART is that approximately half of the myocardial infarctions occurred in individuals without the obstructive coronary disease. Although it is well known that nonobstructive plaques may be responsible for a significant proportion of those events, no study had provided data on its prevalence in lower risk stable individuals until these recent CCTA studies. This finding highlights the need to incorporate the investigation of nonobstructive CAD, regardless of the presence of ischemia (and perhaps symptoms), as those findings can have significant clinical impact and should prompt pharmacological and non-pharmacological interventions.

The recent NICE guidelines from the United Kingdom delineates CCTA as a first line test for the investigation of suspected CAD, regardless of the pretest probability of disease.^13^ The findings from SCOT-HEART, along with the results of Danish registry,^11^ as well as cost-effectiveness analyses^14^ all support the NICE guidelines in its recommendation. Together they provide a consistent and sound body of evidence to challenge the current clinical practice recommendations. As a medical community, we need to embrace these changes and to challenge ourselves whether there is any rationale not to consider CCTA as a first line strategy for the investigation of individuals with suspected obstructive CAD.

## References

[1] Li M, Du XM, Jin ZT, Peng ZH, Ding J, Li L (2014). The diagnostic performance of coronary artery angiography with
64-MSCT and post 64-MSCT: systematic review and
meta-analysis. PloS one.

[2] Stocker TJ, Deseive S, Leipsic J, Hadamitzky M, Chen MY, Rubinshtein R (2018). Reduction in radiation exposure in cardiovascular computed
tomography imaging: results from the Prospective Multicenter Registry on
RadiaTion Dose Estimates of Cardiac CT Angiography IN Daily Practice in 2017
(PROTECTION VI). Eur Heart J.

[3] Agus AM, McKavanagh P, Lusk L, Verghis RM, Walls GM, Ball PA (2016). The cost-effectiveness of cardiac computed tomography for
patients with stable chest pain. Heart.

[4] Hoffmann U, Truong QA, Schoenfeld DA, Chou ET, Woodard PK, Nagurney JT (2012). Investigators R-I: Coronary CT angiography versus standard
evaluation in acute chest pain. N Engl J Med.

[5] Bittencourt MS, Hulten EA, Murthy VL, Cheezum M, Rochitte CE, Di Carli MF (2016). Clinical Outcomes After Evaluation of Stable Chest Pain by
Coronary Computed Tomographic Angiography Versus Usual Care: A
Meta-Analysis. Circ Cardiovasc Imaging.

[6] Douglas PS, Hoffmann U, Patel MR, Mark DB, Al-Khalidi HR, Cavanaugh B (2015). Outcomes of Anatomical versus Functional Testing for Coronary
Artery Disease. N Engl J Med.

[7] Goldstein JA, Chinnaiyan KM, Abidov A, Achenbach S, Berman DS, Hayes SW (2011). Investigators C-S. The CT-STAT (Coronary Computed Tomographic
Angiography for Systematic Triage of Acute Chest Pain Patients to Treatment)
trial. J Am Coll Cardiol.

[8] Hoffmann U, Truong QA, Schoenfeld DA, Chou ET, Woodard PK, Nagurney JT (2012). Coronary CT Angiography versus Standard Evaluation in Acute Chest
Pain. N Engl J Med.

[9] Investigators S-H (2015). CT coronary angiography in patients with suspected angina due to
coronary heart disease (SCOT-HEART): an open-label, parallel-group,
multicentre trial. Lancet.

[10] Fordyce CB, Newby DE, Douglas PS (2016). Diagnostic Strategies for the Evaluation of Chest Pain: Clinical
Implications From SCOT-HEART and PROMISE. J Am Coll Cardiol.

[11] Jørgensen ME, Andersson C, Nørgaard BL, Abdulla J, Shreibati JB, Torp-Pedersen C (2017). Functional Testing or Coronary Computed Tomography Angiography in
Patients With Stable Coronary Artery Disease. J Am Coll Cardiol.

[12] Forbes J, Hunter A, Lewis S, MacLean S, Mills NL, Norrie J (2018). Coronary CT Angiography and 5-Year Risk of Myocardial
Infarction. N Engl J Med.

[13] Moss AJ, Williams MC, Newby DE, Nicol ED (2017). The Updated NICE Guidelines: Cardiac CT as the First-Line Test
for Coronary Artery Disease. Curr Cardiovasc Imaging Rep.

[14] Genders TS, Petersen SE, Pugliese F (2015). The optimal imaging strategy for patients with stable chest pain:
A cost-effectiveness analysis. Ann Intern Med.

